# A Linear Waxy Verrucous Plaque of the Scalp

**DOI:** 10.7759/cureus.37881

**Published:** 2023-04-20

**Authors:** Despoina D Kakagia, Konstantinos C Christodoulou, Christos N Noulas, Ioannis Stouras, Aliki Fiska

**Affiliations:** 1 Department of Plastic Surgery, Democritus University of Thrace, Alexandroupolis, GRC; 2 Laboratory of Anatomy, School of Medicine, Democritus University of Thrace, Alexandroupolis, GRC; 3 First Department of Surgery, University Hospital of Alexandroupolis, Democritus University of Thrace, Alexandroupolis, GRC; 4 Medical School, National and Kapodistrian University of Athens, Athens, GRC

**Keywords:** cutaneous neoplasms, nevus sebaceous of jadassohn, cutaneous hamartoma, organoid nevus, nevus sebaceous

## Abstract

Nevus sebaceous of Jadassohn (NSJ) is an inborn, cutaneous hamartoma that is presented as a round-oval, or linear, yellowish-orange hairless plaque with an excess of sebaceous glands, typically localized to the head or neck. NSJ disease progresses slowly in three general stages. Due to its embryological origin, it yields an already documented potential for a variety of epidermal and adnexal tumors. The incidence of secondary neoplasms within NSJ is 10-30%, and the risk of neoplastic transformation increases with age. The majority of neoplasms are benign. Regarding malignant tumors, NSJ is usually associated with basal cell carcinoma. All neoplasms are typically encountered in long-standing lesions. Owing to NSJ’s ample variety of associations with neoplasms, its management demands a case-driven tailored treatment. We present the case of a 34-year-old female with NSJ.

## Introduction

Considered an epidermal nevus variant, nevus sebaceous (NS) or organoid nevus is an inborn, cutaneous hamartoma first described in 1895 by German dermatologist Josef Jadassohn [1]. Found in up to 1% of newborns, NS is presented as a round-oval, or linear, yellowish-orange hairless plaque with an excess of sebaceous glands. It is primarily localized to the head or neck, along the lines of Blaschko, while its prevalence has also been documented in less common sites such as the trunk, mucosa, and extremities [2]. A more challenging clinical manifestation, known as Schimmelpenning-Feuerstein-Mims or linear nevus syndrome, occurs when NS is accompanied by extracutaneous symptoms, affecting multiple organs [3]. Herein, we report the case of a tricenarian female patient presenting with a NS.

## Case presentation

**Figure 1:**
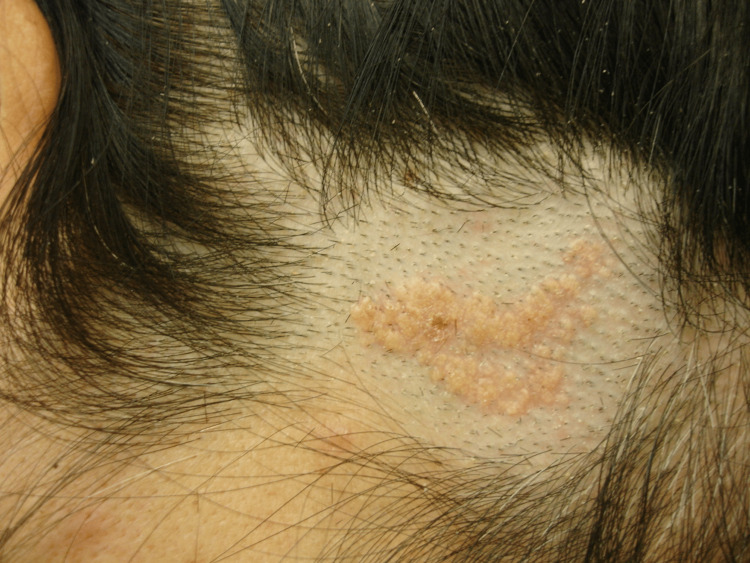
Clinical appearance of the lesion. A yellowish, waxy, hairless, well-defined, L-shaped lesion at the temporal region.

A 34-year-old female patient of Caucasian-Hellenic origin with no prior medical history was referred to the Plastic Surgery Outpatient Department, complaining about an innate verrucous plaque on her scalp that had been discovered since she was born, increased in size in puberty, and had almost doubled in width over the last five years, becoming slightly pruritic. On physical examination, a yellowish, waxy, hairless, well-defined, L-shaped lesion measuring 3.3 cm × 1.4 cm was noticed at the right temporal region (Figure 1). 

**Figure 2:**
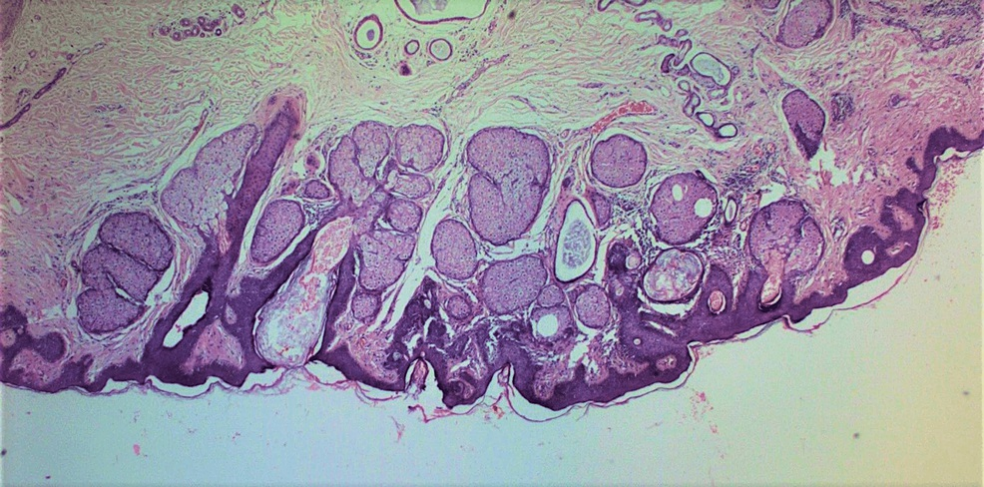
Histopathologic characteristics of the lesion. Hyperkeratosis, focal parakeratosis, papillomatosis, sebaceous gland hyperplasia, and dilated sweat glands with loci of apocrine cells in the deep dermis (H&E X100).

Two weeks after consultation, the patient underwent surgical excision of the lesion with a 3 mm clinical margin. The skin deficit was directly closed. Histopathologic examination revealed hyperkeratosis, focal parakeratosis, papillomatosis, sebaceous gland hyperplasia, and dilated sweat glands with loci of apocrine cells in the deep dermis, without significant mitoses or atypia (Figure 2).

Based on the clinical and histopathologic findings, the diagnosis of a nevus sebaceous of Jadassohn (NSJ) was made. There was no recurrence of the lesion two years after surgery.

## Discussion

NSJ disease progresses slowly in three general stages [2]. Until puberty, NSJ lesions remain flat with small follicular sebaceous glands and may be accompanied by focal alopecia [4]. During adolescence, NSJ thickens and is characterized by papillomatous hyperplasia of the overlying epidermis along with excessive development of the sebaceous and apocrine glands. Ultimately, in some patients above 50 or 60 years of age, the lesion may be susceptible to further changes, leading to the development of benign or malignant tumors on top of the original nevus [2,4].

Since NSJ presumably originated from pluripotent primary epithelial germ cells, it yields an already documented potential towards a variety of epidermal and adnexal tumors [5]. In addition, its androgen receptor activity and expression of post-zygotic mutations in the Harvey rat sarcoma viral oncogene homolog (HRAS) and Kristen rat sarcoma viral oncogene homolog (KRAS) genes may initiate oncogenesis [4]. The incidence of secondary neoplasms within NSJ is 10-30%, and the risk of neoplastic transformation increases with age [5]; hence caution must be exerted in case of any change in the appearance of the lesion [2]. The overwhelming majority of neoplasms are benign, with less than 1% being malignant, with trichoblastoma and syringocystadenoma papilliferum, followed by trichilemmoma and sebaceoma, being the most frequently documented benign tumors [4]. Regarding malignant tumors, NSJ is usually associated with basal cell carcinoma, while squamous cell carcinoma, keratoacanthoma, and sebaceous carcinoma have also been reported [2]. All neoplasms are typically encountered in long-standing lesions [6], while multiple neoplasms may occasionally arise within the same lesion. However, simultaneously presentation of malignancies is rare [5].

The diagnosis of NSJ is usually clinical and confirmed by histopathologic examination; large sebaceous glands associated with hyperkeratosis, papillomatosis, acanthosis with hypergranulosis, defective hair follicles, and heterotopic apocrine glands are the typical histologic features. The differential diagnosis of NSJ includes entities such as colliding xanthogranulomas (which generally develop rapidly into distinctive domed, papular, or nodular lesions), epidermodysplasia verruciformis (which mainly manifests as multiple verrucous cutaneous lesions), seborrheic keratosis (which mainly manifests as multiple verrucous cutaneous lesions), congenital nevus (which is typically pigmented), syringocystadenoma papilliferum (with its surface pinkish and nodular rather than yellow and velvety), and solitary mastocytoma (with infiltration of primarily the papillary dermis by mast cells and the presence of Darier's sign, which is pathognomonic). Histopathologic examination clearly identifies all the aforementioned disorders. [4]. Owing to NSJ’s ample variety of associations with neoplasms, its management demands a case-driven tailored treatment [4]. Subsequently, in a benign lesion, prophylactic excision is recommended [2], while for NSJ-related malignancies Mohs micrographic surgery or wide surgical excision is considered the definitive treatment, diminishing complications and tumor recurrence [4].

## Conclusions

In conclusion, NSJ is a rare, congenital skin lesion that necessitates close monitoring as it may be the precursor to various malignancies. Even though its precise pathophysiology is yet to be unveiled, early detection and tailored treatment can have a positive effect on a patient’s outcome. Physicians should always have a high level of awareness for optimal treatment to be achieved.
